# The effect of *Lactobacillus* with prebiotics on KPC-2-producing *Klebsiella pneumoniae*

**DOI:** 10.3389/fmicb.2022.1050247

**Published:** 2022-12-07

**Authors:** Hung-Jen Tang, Chi-Chung Chen, Ying-Chen Lu, Hui-Ling Huang, Hung-Jui Chen, Yin-Ching Chuang, Chih-Cheng Lai, Chien-Ming Chao

**Affiliations:** ^1^Department of Internal Medicine, Chi Mei Medical Center, Tainan, Taiwan; ^2^Department of Medical Research, Chi Mei Medical Center, Tainan, Taiwan; ^3^Department of Food Science, National Chiayi University, Chiayi, Taiwan; ^4^Division of Hospital Medicine, Department of Internal Medicine, Chi Mei Medical Center, Tainan, Taiwan; ^5^Department of Intensive Care Medicine, Chi Mei Medical Center, Liouying, Tainan, Taiwan

**Keywords:** KPC, *Klebsiella pneumoniae*, probiotic, prebiotic, synbiotic

## Abstract

**Objectives:**

This study investigated the inhibitory effect of *Lactobacillus* spp. with prebiotics against *Klebsiella pneumoniae* carbapenemase-2 (KPC-2)-producing *Klebsiella pneumoniae* using both *in vitro* experiments and animal models.

**Methods:**

Thirty-three *Lactobacillus* spp. strains were confirmed by 16S rDNA sequencing, and four different PFGE genotyped KPC-2-producing *K. pneumoniae* strains were selected for investigation. *In vitro* studies, including broth microdilution assays, changes in pH values in lactobacilli cultures with different prebiotics, time-kill tests of *Lactobacillus* spp. against KPC-2-producing *K. pneumoniae* and further *in vivo Lactobacillus* alone or in combination with prebiotics against KPC-2-producing *K. pneumoniae* in an animal model, were performed.

**Results:**

The lower pH value of the cell-free supernatant was associated with a lower minimal inhibitory percentage of the *Lactobacillus* strain against KPC-2-producing *K. pneumoniae.* Furthermore, lactulose/isomalto-oligosaccharide/inulin and fructo-oligosaccharide can enhance the inhibitory effect of all 10^7^ CFU/ml *Lactobacillus* strains against KPC001. Three *Lactobacillus* strains (LYC1154, LYC1322, and LYC1511) that could be persistently detected in the stool were tested for their ability to reduce the amount of KPC001 in the feces individually or in combination. A significantly better effect in reducing the amount of KPC001 was observed for the combination of three different *Lactobacillus* species than for each of them alone. Furthermore, their inhibitory effect was enhanced after adding lactulose or isomalto-oligosaccharide (both *p* < 0.05).

**Conclusion:**

This study demonstrates the inhibitory effect of probiotic *Lactobacillus*, including LYC1154, LYC1322, and LYC1511, with prebiotics such as lactulose or isomalto-oligosaccharide against the colonization of KPC-2-producing *K. pneumoniae*.

## Introduction

Probiotics are living bacteria or fungi that are consumed, and prebiotics are nondigestible compounds that are selectively fermented by commensal microbiota in the human gut (Gibson and Roberfroid, 1995; Newman and Arshad, 2020). Both probiotics and prebiotics can provide health benefits to the host and promote human health. Probiotics as well as prebiotics exert their effect through the production of short-chain fatty acids from metabolic precursors, leading to the downstream effects of immune modulation and increased mucosal barrier function (Patel and DuPont, 2015). Moreover, probiotics with or without prebiotics can exhibit the additional effect of producing antimicrobial compounds and restoring the enteric microbiome (Patel and DuPont, 2015; Newman and Arshad, 2020). In fact, several commercial probiotics have been used to manage *Clostridioides difficile* infections, traveler’s diarrhea, and irritable bowel syndrome (Sniffen et al., 2018). Moreover, the use of prebiotics, probiotics and synbiotics shows promising potential in the manipulation of the microbiome and resistome and helps combat MDROs (Newman and Arshad, 2020).

Antibiotics are essential in the treatment of acute bacterial infections; however, overuse or misuse of antibiotics in humans, agriculture, and animal husbandry has resulted in the emergence of a wide range of multidrug-resistant organisms (MDROs) (Dadgostar, 2019). Among MDROs, carbapenem-resistant gram-negative bacteria, including carbapenem-resistant *Enterobacterales* (CREs), *Acinetobacter baumannii* and *Pseudomonas aeruginosa*, have become the common cause of health care-associated infections and pose a great threat to global health (Dadgostar, 2019; Lai and Yu, 2021; Wozniak et al., 2022; Jean et al., 2022b). Both the World Health Organization and Centers for Disease Control and Prevention have designated that CRE, such as *Klebsiella* species, *Escherichia coli* and *Enterobacter* species, are the most crucial emerging resistance threats worldwide (Hetzler et al., 2022; Lodise et al., 2022; Oka et al., 2022; Sy et al., 2022; Jean et al., 2022a). The most common resistance mechanism of carbapenem resistance in *Enterobacterales* is the synthesis of carbapenemase enzymes, which include class A *Klebsiella pneumoniae* carbapenemase (KPC), class B metallo-β-lactamases, and class D OXA β-lactamases (Durante-Mangoni et al., 2019). Because these bacteria are difficult to treat due to high levels of antibiotic resistance and the decreased focus of pharmaceutical industries on research and development of newer effective antibiotics to fight these MDROs, novel strategies such as nanoparticles, phage therapy, antimicrobial peptides, and fecal microbiota transplantation are urgently needed to fight these MDROs (Sannathimmappa et al., 2021).

In our previous *in vitro* studies using agar well diffusion and broth microdilution assays and time-kill tests, we demonstrated the potent activity of *Lactobacillus* spp. against CRE and carbapenemase-producing *Enterobacterales* (Chen et al., 2019, 2021). Furthermore, we found that lactic acid produced by *Lactobacillus* strains is the major antimicrobial mechanism. However, we considered whether adding prebiotics with *Lactobacillus* spp. would help enhance their activity against these MDROs. Therefore, we conducted this study to assess the inhibitory effect of *Lactobacillus* spp. with prebiotics against KPC-2-producing *K. pneumoniae* using both *in vitro* experiments and animal models.

## Materials and methods

### Bacterial strains

Thirty-three *Lactobacillus* spp. strains were isolated from Chinese sauerkraut as confirmed by 16S rDNA sequencing. The 16S rRNA gene was amplified with primers F27 (AGAGTTTGATCM TGGCTCAG) and R1492 (TACGGYTACCTTGTTACGACTT) as previously reported (Cruciani et al., 2015). The sequences were searched with the NCBI BLAST web service (blast.ncbi.nlm.nih.gov) to confirm the taxonomic identification at the species level. Strains including 13 *L. plantarum*, 8 *L. paracasei*, 7 *L. fermentum*, 4 *L. rhamnosus*, and 1 *L. brevis* were selected. The basic growth medium for *Lactobacillus* spp. was Man-Rogosa-Sharpe (MRS; Oxoid Inc., Ogdensburg, NY, United States). Four different pulse field gel electrophoresis (PFGE)-genotyped KPC-2-producing *K. pneumonia* strains were selected with a CHEF DR II apparatus (Bio-Rad Laboratories, Hercules, CA, United States) (Tang et al., 2019).

### Cell-free supernatant preparation

The culture supernatants of lactobacilli were grown in MRS broth (pH adjusted to 6.5) at 37°C for 24 h. The culture broths were then centrifuged at 10,000*g* at 4°C for 30 min. The supernatants were sterilized by filtration through a 0.22 μm cellulose acetate filter (Millipore, Billerica, MA, United States) and stored at −80°C until use.

### Broth microdilution assay

A broth microdilution assay was conducted as previously described with modifications (Śliżewska and Chlebicz-Wójcik, 2020). Overnight cultures of KPC-2-producing *K. pneumoniae* were inoculated into fresh Mueller-Hinton broth (MHB) media and seeded into 96-well plates (BD Discovery Labware, Bedford, MA, United States). The CFSs were diluted with MRS broth (pH = 6.5) and used at different percentages (i.e., 6.25%, 12.5%, 25%, and 50%) in the final 200 μl volume. The minimum inhibitory percentage (MIP), defined as the lowest percentage of supernatant that can inhibit the growth of pathogens, was monitored by measuring the optical density (OD600 nm). All tests were performed in triplicate.

### The effect on pH value by *Lactobacillus* and different prebiotics

The effect of *Lactobacillus* spp. strains after adding prebiotics, including inulin (IN), fructooligosaccharide (FOS), and lactulose (LU) (Sigma–Aldrich, St. Louis, MO, United States), isomalto-oligosaccharide (IMO) (FUJIFILM, Chuo-Ku, Osaka, Japan), and xylo-oligosaccharide (XOS) (Americanway Bio-Technology, Tainan, Taiwan), on pH value was analyzed. *Lactobacillus* spp. were added as 10^4^ CFU/ml inoculum to MRS broths in which glucose was substituted with 2% prebiotic or sucrose (Sigma–Aldrich, St. Louis, MO, United States; Lu et al., 2018; Śliżewska and Chlebicz-Wójcik, 2020; Chen et al., 2021). The cultures were incubated for 24 h at 37°C. The pH values were measured using a pH meter. All tests were performed in triplicate.

### Time-kill test of *Lactobacillus* spp. against KPC-2-producing *Klebsiella pneumoniae*

First, KPC-2-producing *K. pneumoniae* and *Lactobacillus* strains were individually cultured in their own broth medium at 37°C for 24 h. The cultures were centrifuged at 6,000 rpm and 22°C for 10 min to collect the cell pellet. Second, KPC-2-producing *K. pneumoniae* were inoculated at 1 × 10^6^ CFU/ml and cocultured with 1 × 10^5^, 1 × 10^6^, or 1 × 10^7^ CFU/ml lactobacilli in tubes containing 10 ml of MRS-MH broth (1:1) with 2% different probiotics, sucrose as a control or no carbohydrate at 37°C for 48 h (Drago et al., 1997). Samples were collected at 0, 3, 6, 24, and 48 h for the determination of viable cell count and pH measurements. A 1 ml aliquot of each sample was used to prepare serial dilutions that were poured onto the appropriate agar plates; MRS agar (pH 5.5) was used for *Lactobacillus* spp., while EMB agar (Oxoid Inc., Ogdensburg, NY, United States) + 16 μg/ml ampicillin (Sigma–Aldrich, St. Louis, MO, United States) was used for pathogen plates that were incubated at 37°C for 24 h, and colonies were counted. All tests were performed in triplicate.

### Antimicrobial treatment protocol to eradicate the intestinal pathogen in mice

This animal study was approved by the Institutional Animal Care and Use Committee of Chi Mei Medical Center (IACUC Approval No 108101803). Animals were housed under controlled temperatures and 12 h/12 h light/dark cycles with *ad libitum* access to food and water. Before the study, the mice were allowed to adapt for the last 5 days. The humane endpoint was set to a loss of 20% body weight compared with the starting weight in each experiment. Female inbred BALB/c mice (Animal Center, National Science Council, Taipei, Taiwan) weighing 18–20 g (6–8 weeks old) were used in this study. For every animal, antimicrobials started with 3 days of 0.1 mg/ml amphotericin-B for 0.1 ml by gavage every 12 h. In addition, water flasks were supplemented with 1 g/L ampicillin (Bristol Meyers Squibb, New York City, NY), 5 mg/ml vancomycin, 10 mg/ml neomycin, and 10 mg/ml metronidazole. All drugs were purchased from Sigma–Aldrich (Sigma–Aldrich, St. Louis, MO, United States). Fresh antibiotics were added daily into drinking water for three consecutive days (Reikvam et al., 2011). After 3 days of antibiotic treatment, the fresh feces of each animal were collected directly into a preweighed 1.5 ml capped microtube. Tubes with fecal pellets were kept on ice and weighed, and the weight of the pellets was calculated. Fecal pellets were resuspended in 1 ml PBS by vortexing. The fecal suspension was then plated on blood agar, anaerobic blood agar, and yeast agar (Sabouraud agar) with 100 μl suspension on each plate. Blood agar and Sabouraud agar plates were incubated aerobically at 37°C with 5% CO_2_ for 72 h, while anaerobic blood agar plates were incubated at 37°C in anaerobic conditions for 72 h. At the end of incubation, the numbers of colonies on the plates were counted to confirm whether the fecal bacteria were eradicated. The detection limit of the assay was defined as 1 cfu/mg feces (Reikvam et al., 2011; Jeong et al., 2017).

### *Lactobacillus* colonization test

**Figure 1:**
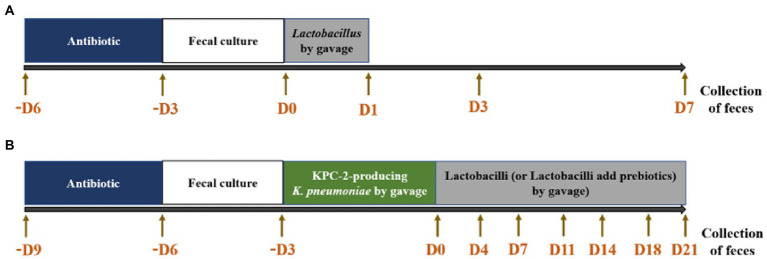
Flow chart of the animal assay.

After 3 days of antimicrobial treatment, no microbes were detected in the animal feces. A total of 2 × 10^9^ CFU/animal of each *Lactobacillus* strain, including 6 *L. plantarum*, 3 *L. paracasei*, and 3 *L. rhamnosus*, resuspended in PBS was added individually to the gavage tube. On Days 1, 3, and 7 after adding lactobacilli, fresh feces were collected. The fecal suspensions were serially diluted 10-fold, plated on MRS agar and incubated at 37°C for 48 h. After incubation, the numbers of colonies on the plates (MRS pH 5.0 + 32 μg/ml vancomycin) were counted, and the number of *Lactobacillus* spp. per g of feces was calculated (Figure 1A).

### *Lactobacillus* combined with prebiotics against KPC-2-producing *Klebsiella pneumoniae* in an animal model

After 3 days of antimicrobial treatment and animal feces without detectable microbes, 3 × 10^8^ CFU of KPC-2-producing *K. pneumoniae* was added by oral gavage for 3 days for every animal. On the day after the last pathogen treatment, LYC1154, LYC1322, and LYC1511 (2 × 10^9^ CFU each *Lactobacillus*/animal/200 μl) combined with 20% LU, IMO, or PBS alone were added daily until the end of the study. Feces were collected on Days 0 (1 h before *Lactobacillus* treatment), 4, 7, 11, 14, 18, and 21 (Figure 1B). *Lactobacillus* was detected by MRS agar (pH = 5.0) + 32 μg/ml vancomycin, and KPC-2-producing *K. pneumoniae* was detected by EMB agar with 16 μg/ml eartapenem + 64 μg/ml ampicillin + 16 μg/ml cefotaxime.

### *Lactobacillus* alone or in combination with three strains against KPC-2-producing *Klebsiella pneumoniae* in mice

LYC1154, LYC1322, and LYC1511 were treated alone at 6 × 10^9^ CFU in PBS. The combination of LYC1154, LYC1322, and LYC1511 included 2 × 10^9^ CFU per strain. Antibiotic treatment and KPC-2-producing *K. pneumoniae* infection were performed as described above. LYC1154, LYC1322, and LYC1511 were treated alone at 6 × 10^9^ CFU in PBS. Alternatively, LYC1154, LYC1322, and LYC1511 were combined (2 × 10^9^ CFU per strain). The fecal treatments are described above.

#### Statistical analysis

When appropriate, data are presented as the mean and standard deviation (mean ± SD). The two-tailed *t*-test was used for statistical analysis. The *p*-value for statistical significance for all analyses was defined as *p* < 0.05.

## Results

### The minimal inhibition percentage against the CPE and pH of lactobacilli CFS

**Figure 2:**
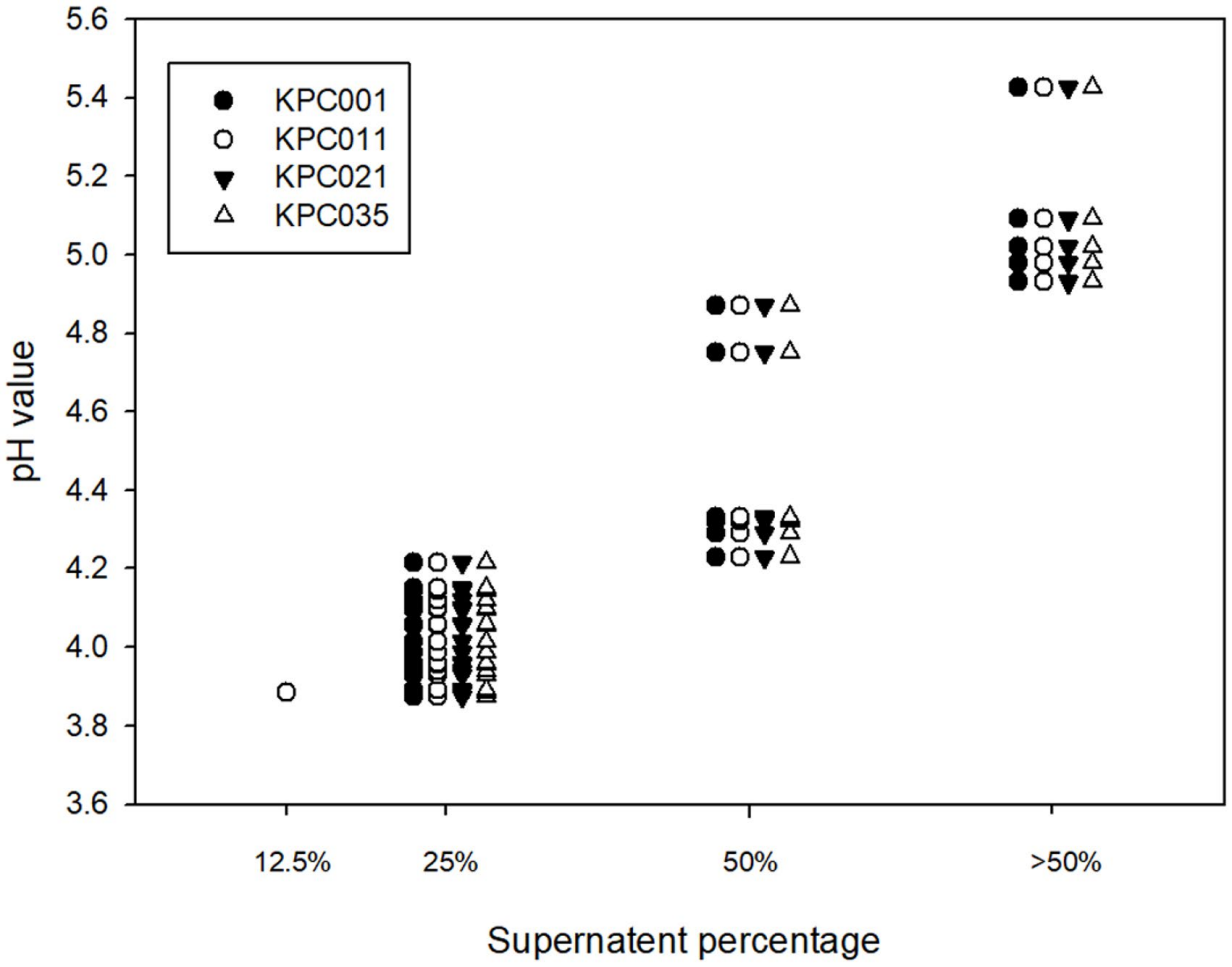
Correlation of the pH of cell-free supernatants and MIP against carbapenemase-producing *Klebsiella pneumoniae*.

The pH of *Lactobacillus* strain CFS varied according to different *Lactobacillus* species (Table 1). The resulting pH value was lowest in the CFS of *L. plantarum*, followed by *L. paracasei*, *L. fermentum*, and *L rhamnosus*. Furthermore, the lower pH value of CFS was associated with a lower MIP of the *Lactobacillus* strain against KPC-2-producing *K. pneumoniae,* and this finding was consistent across 4 KPC-2-producing *K. pneumoniae* strains (Table 1 and Figure 2). Then, 28 *Lactobacillus* strains with MIP ≤ 50% were selected for the following tests with prebiotics.

**Table 1:** The resulting pH and MIPs of *Lactobacillus* strain cell-free supernatants (%) against KPC-2-producing *Klebsiella pneumoniae.*

No.	Species	pH	Minimal inhibition percentage			
		Mean ± SD	KPC001	KPC011	KPC021	KPC035
LYC1322	*L. plantarum*	3.88 ± 0.05	25%	25%	25%	25%
LYC1031	*L. plantarum*	3.89 ± 0.03	25%	12.5%	25%	25%
LYC1143	*L. plantarum*	3.89 ± 0.07	25%	25%	25%	25%
LYC1159	*L. plantarum*	3.93 ± 0.08	25%	25%	25%	25%
LYC1112	*L. plantarum*	3.94 ± 0.07	25%	25%	25%	25%
LYC1115	*L. plantarum*	3.96 ± 0.04	25%	25%	25%	25%
LYC1141	*L. plantarum*	3.96 ± 0.09	25%	25%	25%	25%
LUC0289	*L. plantarum*	3.96 ± 0.08	25%	25%	25%	25%
LYC1154	*L. paracasei*	3.96 ± 0.10	25%	25%	25%	25%
LYC1146	*L. plantarum*	3.99 ± 0.06	25%	25%	25%	25%
LYC1138	*L. plantarum*	4.02 ± 0.08	25%	25%	25%	25%
LYC1117	*L. plantarum*	4.06 ± 0.04	25%	25%	25%	25%
LYC1088	*L. plantarum*	4.06 ± 0.08	25%	25%	25%	25%
LUC0219	*L. plantarum*	4.10 ± 0.11	25%	25%	25%	25%
LYC1151	*L. paracasei*	4.10 ± 0.14	25%	25%	25%	25%
LUC0040	*L. paracasei*	4.12 ± 0.06	25%	25%	25%	25%
LYC1149	*L. paracasei*	4.15 ± 0.09	25%	25%	25%	25%
LYC1229	*L. paracasei*	4.15 ± 0.11	25%	25%	25%	25%
LYC1119	*L. paracasei*	4.22 ± 0.07	25%	25%	25%	25%
LUC0182	*L. fermentum*	4.23 ± 0.09	50%	50%	50%	50%
LYC1504	*L. rhamnosus*	4.26 ± 0.08	50%	50%	50%	50%
LYC1511	*L. rhamnosus*	4.27 ± 0.09	50%	50%	50%	50%
LUC0191	*L. fermentum*	4.29 ± 0.08	50%	50%	50%	50%
LYC1120	*L. fermentum*	4.32 ± 0.09	50%	50%	50%	50%
LUC0168	*L. fermentum*	4.33 ± 0.05	50%	50%	50%	50%
LUC0174	*L. fermentum*	4.33 ± 0.04	50%	50%	50%	50%
LUC0127	*L. rhamnosus*	4.75 ± 0.07	50%	50%	50%	50%
LUC0413	*L. rhamnosus*	4.87 ± 0.06	50%	50%	50%	50%
LYC1142	*L. paracasei*	4.93 ± 0.16	>50%	>50%	>50%	>50%
LYC1118	*L. rhamnosus*	4.98 ± 0.14	>50%	>50%	>50%	>50%
LYC1065	*L. rhamnosus*	5.02 ± 0.04	>50%	>50%	>50%	>50%
LYC1152	*L. brevis*	5.09 ± 0.08	>50%	>50%	>50%	>50%
LUC0180	*L. paracasei*	5.43 ± 0.16	>50%	>50%	>50%	>50%

### pH value in *Lactobacillus* cultures with different prebiotics

Overall, the pH value in the 19/28 *Lactobacillus* strains cultured with LU was lower than that in those cultured with other prebiotics. Among 13 *L. plantarum* strains, LYC1031, L1112, L1117, LYC1146, LYC1159, and LYC1322 could be associated with lower pH after adding LU than other strains (Table 2). Among the 6 *L. paracasei* strains, LYC1119, LYC1154, and LYC1229 had lower pH values with LU. Among the 4 *L. rhamnosus* strains, the pH was lower in the coculture with LYC1504 and LYC15111. However, a lower pH in the coculture with prebiotics was not observed for the *L. fermentum* strains. Therefore, 11 *Lactobacillus* strains, including LYC1031, L1112, L1117, LYC1146, LYC1159, LYC1322, LYC1119, LYC1154, LYC1229, LYC1504, and LYC1511, which could produce lower pH values with prebiotics, were selected for testing with prebiotics against KPC-2-producing *K. pneumoniae.*

**Table 2:** The pH value of *Lactobacillus* strains cultured with MRS with different carbohydrates.

*L. plantarum*	Carbohydrate	LUC0219	LUC0289	LYC1031	LYC1088	LYC1112	LYC1115	LYC1117
	Non	6.19 ± 0.01	6.13 ± 0.00	6.19 ± 0.00	6.02 ± 0.00	6.06 ± 0.01	6.15 ± 0.01	6.15 ± 0.00
	SUC	4.21 ± 0.03	3.99 ± 0.03	3.83 ± 0.05	3.99 ± 0.05	3.95 ± 0.04	3.94 ± 0.04	4.01 ± 0.04
	FOS	5.89 ± 0.02	5.88 ± 0.02	5.94 ± 0.01	5.87 ± 0.03	5.81 ± 0.01	4.99 ± 0.03	3.78 ± 0.06
	IN	5.50 ± 0.01	5.04 ± 0.01	4.97 ± 0.01	4.95 ± 0.02	4.88 ± 0.03	4.81 ± 0.02	3.77 ± 0.04
	IMO	4.68 ± 0.04	4.29 ± 0.02	4.48 ± 0.02	4.53 ± 0.03	4.37 ± 0.03	4.38 ± 0.01	3.91 ± 0.05
	LU	4.07 ± 0.02	3.85 ± 0.04	3.74 ± 0.04	3.95 ± 0.04	3.79 ± 0.06	3.82 ± 0.03	3.72 ± 0.03
	XOS	5.21 ± 0.01	5.05 ± 0.01	5.15 ± 0.01	4.98 ± 0.02	5.09 ± 0.02	5.03 ± 0.02	5.09 ± 0.02
	**Carbohydrate**	**LYC1138**	**LYC1141**	**LYC1143**	**LYC1146**	**LYC1159**	**LYC1322**	
	Non	6.20 ± 0.00	6.13 ± 0.00	6.10 ± 0.00	6.13 ± 0.01	6.19 ± 0.01	6.21 ± 0.00	
	SUC	3.94 ± 0.06	4.18 ± 0.03	3.83 ± 0.04	3.99 ± 0.04	3.98 ± 0.05	3.82 ± 0.05	
	FOS	5.86 ± 0.02	5.89 ± 0.01	5.91 ± 0.02	3.69 ± 0.06	3.70 ± 0.06	5.94 ± 0.01	
	IN	5.07 ± 0.02	4.95 ± 0.02	5.00 ± 0.02	3.80 ± 0.05	3.72 ± 0.04	5.03 ± 0.01	
	IMO	3.96 ± 0.05	4.25 ± 0.03	4.60 ± 0.03	4.58 ± 0.03	4.45 ± 0.03	4.57 ± 0.03	
	LU	3.89 ± 0.05	3.93 ± 0.06	3.84 ± 0.06	3.72 ± 0.04	3.76 ± 0.04	3.78 ± 0.04	
	XOS	5.12 ± 0.02	5.04 ± 0.03	5.18 ± 0.01	5.19 ± 0.02	5.18 ± 0.02	5.17 ± 0.01	
***L. paracasei***	**Carbohydrate**	**LUC0040**	**LYC1119**	**LYC1149**	**LYC1151**	**LYC1154**	**LYC1229**	
	Non	6.14 ± 0.01	6.20 ± 0.00	6.15 ± 0.00	6.07 ± 0.01	6.12 ± 0.01	6.14 ± 0.00	
	SUC	5.70 ± 0.02	5.47 ± 0.02	5.47 ± 0.03	5.81 ± 0.01	3.82 ± 0.03	4.34 ± 0.02	
	FOS	6.11 ± 0.02	4.10 ± 0.03	6.07 ± 0.00	6.06 ± 0.00	3.68 ± 0.04	4.07 ± 0.02	
	IN	5.66 ± 0.02	4.09 ± 0.02	5.74 ± 0.03	5.72 ± 0.02	3.70 ± 0.05	4.04 ± 0.04	
	IMO	4.68 ± 0.03	5.10 ± 0.02	5.03 ± 0.02	5.01 ± 0.02	4.60 ± 0.02	4.61 ± 0.02	
	LU	5.17 ± 0.02	4.36 ± 0.05	5.40 ± 0.03	5.72 ± 0.01	3.94 ± 0.04	4.37 ± 0.03	
	XOS	5.30 ± 0.01	5.56 ± 0.02	5.41 ± 0.03	5.39 ± 0.03	5.02 ± 0.03	5.34 ± 0.00	
***L. rhamnosus***	**Carbohydrate**	**LUC 0127**	**LUC 0413**	**LYC1504**	**LYC1511**			
	Non	6.06 ± 0.01	6.10 ± 0.00	6.13 ± 0.02	6.08 ± 0.02			
	SUC	5.99 ± 0.02	6.05 ± 0.02	5.29 ± 0.01	5.31 ± 0.01			
	FOS	6.05 ± 0.01	6.11 ± 0.01	5.85 ± 0.03	5.89 ± 0.07			
	IN	5.72 ± 0.03	5.80 ± 0.03	5.41 ± 0.03	5.40 ± 0.01			
	IMO	5.02 ± 0.04	5.49 ± 0.00	4.28 ± 0.01	4.29 ± 0.01			
	LU	5.31 ± 0.03	6.10 ± 0.01	3.83 ± 0.01	3.78 ± 0.02			
	XOS	5.45 ± 0.02	5.73 ± 0.02	5.18 ± 0.02	5.21 ± 0.01			
***L. fermentum***	**Carbohydrate**	**LUC0168**	**LUC00174**	**LUC0182**	**LUC0191**	**LYC1120**		
	Non	6.01 ± 0.02	6.20 ± 0.01	6.22 ± 0.00	6.23 ± 0.01	6.21 ± 0.01		
	SUC	4.32 ± 0.03	4.48 ± 0.03	4.58 ± 0.02	4.47 ± 0.02	5.27 ± 0.02		
	FOS	5.69 ± 0.01	5.85 ± 0.04	6.09 ± 0.02	6.09 ± 0.00	5.63 ± 0.01		
	IN	5.51 ± 0.03	5.52 ± 0.02	5.84 ± 0.01	5.49 ± 0.01	5.37 ± 0.03		
	IMO	4.63 ± 0.01	4.66 ± 0.01	4.66 ± 0.02	4.66 ± 0.04	5.10 ± 0.02		
	LU	4.12 ± 0.03	4.06 ± 0.05	5.11 ± 0.01	4.13 ± 0.04	5.45 ± 0.02		
	XOS	5.79 ± 0.05	5.94 ± 0.02	6.05 ± 0.03	5.89 ± 0.01	5.70 ± 0.01		

Non, no added carbohydrate; SUC, sucrose; FOS, fructooligosaccharide; IN, inulin; IMO, isomalto-oligosaccharide; LU, lactulose; XOS, xylooligosaccharide.

### Time-kill test of *Lactobacillus* spp. with prebiotics against KPC-2-producing *Klebsiella pneumoniae*

For all 11 *Lactobacillus* strains tested, LU enhanced the inhibitory effect against KPC001 (Table 3). IMO helped enhance the inhibitory effect of 10 *Lactobacillus* strains, including LYC1031, LYC1112, LYC1117, LYC1146, LYC1159, LYC1322, LYC1154, LYC1229, LYC1504, and LYC1511. IN and FOSs were found to enhance the inhibitory effect of 7 *Lactobacillus* strains, including LYC1117, LYC1146, LYC1159, LYC1119, LYC1154, LYC1229, and LYC1511. However, no inhibitory effect was observed for *Lactobacillus* strains with xylo-oligosaccharide.

**Table 3:** The change in log10 CFU/ml of KPC001 cocultured with *Lactobacillus* strains in MRS-MH broth with different carbohydrates at 24 **(A)** and 48 **(B)** hours.

Carbohydrate	CFU/ml	Non	SUC	FOS	IN	IMO	LU	XOS
**(A)**								
LYC1031	10^5^	3.03 ± 0.18	−1.13 ± 0.22	2.49 ± 0.28	2.83 ± 0.08	2.46 ± 0.16	−1.17 ± 0.32	2.94 ± 0.13
	10^6^	3.01 ± 0.16	−6.17 ± 0.00*	2.98 ± 0.18	2.43 ± 0.18	1.24 ± 0.26	−6.17 ± 0.00*	2.83 ± 0.08
	10^7^	2.55 ± 0.26	−6.17 ± 0.00*	2.55 ± 0.20	1.87 ± 0.26	1.15 ± 0.38	−6.17 ± 0.00*	2.24 ± 0.18
LYC1112	10^5^	2.93 ± 0.12	−1.25 ± 0.41	3.05 ± 0.09	3.08 ± 0.05	2.39 ± 0.10	0.17 ± 0.52	3.17 ± 0.08
	10^6^	2.80 ± 0.23	−3.30 ± 0.30	3.03 ± 0.08	2.89 ± 0.10	1.80 ± 0.35	−6.15 ± 0.00*	2.75 ± 0.15
	10^7^	2.85 ± 0.14	−6.15 ± 0.00*	2.75 ± 0.13	1.89 ± 0.22	−0.07 ± 0.42	−6.15 ± 0.00*	2.37 ± 0.06
LYC1117	10^5^	3.03 ± 0.13	−1.04 ± 0.47	−6.15 ± 0.00*	−6.15 ± 0.00*	1.36 ± 0.38	−1.11 ± 0.55	3.17 ± 0.09
	10^6^	3.00 ± 0.11	−6.15 ± 0.00*	−6.15 ± 0.00*	−6.15 ± 0.00*	−0.04 ± 0.50	−6.15 ± 0.00*	3.17 ± 0.08
	10^7^	2.89 ± 0.25	−6.15 ± 0.00*	−6.15 ± 0.00*	−6.15 ± 0.00*	−6.15 ± 0.00*	−6.15 ± 0.00*	2.99 ± 0.16
LYC1119	10^5^	3.40 ± 0.14	4.23 ± 0.08	2.38 ± 0.16	2.42 ± 0.09	3.42 ± 0.09	2.25 ± 0.23	3.85 ± 0.09
	10^6^	3.37 ± 0.09	2.15 ± 0.33	1.31 ± 0.29	1.56 ± 0.46	3.29 ± 0.07	2.25 ± 0.26	3.43 ± 0.13
	10^7^	3.29 ± 0.17	2.35 ± 0.30	−5.03 ± 0.00*	−5.03 ± 0.00*	2.38 ± 0.16	1.17 ± 0.45	3.33 ± 0.06
LYC1146	10^5^	2.82 ± 0.16	−1.30 ± 0.34	0.62 ± 0.34	−0.47 ± 0.56	1.59 ± 0.16	−5.79 ± 0.00*	3.16 ± 0.06
	10^6^	3.25 ± 0.06	−2.38 ± 0.44	−5.79 ± 0.00*	−1.64 ± 0.34	0.61 ± 0.44	−5.79 ± 0.00*	2.59 ± 0.16
	10^7^	2.73 ± 0.24	−5.79 ± 0.00*	−5.79 ± 0.00*	−5.79 ± 0.00*	−0.59 ± 0.24	−5.79 ± 0.00*	2.29 ± 0.11
LYC1154	10^5^	2.51 ± 0.25	3.20 ± 0.09	1.48 ± 0.30	2.53 ± 0.25	3.07 ± 0.10	2.37 ± 0.37	3.03 ± 0.17
	10^6^	2.54 ± 0.17	0.52 ± 0.43	−2.59 ± 0.23	0.56 ± 0.39	2.48 ± 0.27	−2.62 ± 0.25	2.57 ± 0.19
	10^7^	2.41 ± 0.27	−1.85 ± 0.33	−6.08 ± 0.00*	−0.46 ± 0.12	0.55 ± 0.13	−3.00 ± 0.22	2.32 ± 0.27
LYC1159	10^5^	3.73 ± 0.08	1.77 ± 0.23	3.08 ± 0.07	0.47 ± 0.33	3.04 ± 0.07	0.04 ± 0.19	3.55 ± 0.09
	10^6^	3.50 ± 0.17	0.74 ± 0.39	1.50 ± 0.35	0.47 ± 0.27	1.96 ± 0.37	−0.17 ± 0.23	3.08 ± 0.15
	10^7^	3.52 ± 0.07	−5.68 ± 0.00*	−5.68 ± 0.00*	−3.38 ± 0.23	−0.50 ± 0.35	−5.68 ± 0.00*	2.92 ± 0.09
LYC1229	10^5^	2.52 ± 0.20	3.21 ± 0.02	1.49 ± 0.31	2.47 ± 0.18	2.49 ± 0.10	2.38 ± 0.08	2.99 ± 0.09
	10^6^	2.60 ± 0.18	1.18 ± 0.38	0.60 ± 0.37	−0.48 ± 0.40	1.43 ± 0.35	1.36 ± 0.20	2.54 ± 0.14
	10^7^	2.52 ± 0.09	0.48 ± 0.40	−0.59 ± 0.49	−6.05 ± 0.00*	−0.46 ± 0.22	0.57 ± 0.44	2.35 ± 0.18
LYC1322	10^5^	3.43 ± 0.08	0.32 ± 0.37	3.32 ± 0.13	3.43 ± 0.15	2.81 ± 0.13	−0.54 ± 0.39	3.28 ± 0.13
	10^6^	3.00 ± 0.18	0.62 ± 0.53	3.05 ± 0.13	3.46 ± 0.13	1.86 ± 0.28	0.66 ± 0.47	2.91 ± 0.27
	10^7^	3.46 ± 0.09	−5.72 ± 0.00*	3.46 ± 0.09	2.23 ± 0.23	−5.72 ± 0.00*	−5.72 ± 0.00*	2.60 ± 0.29
LYC1504	10^5^	3.08 ± 0.13	2.96 ± 0.15	3.01 ± 0.11	3.07 ± 0.11	3.08 ± 0.13	2.92 ± 0.16	3.08 ± 0.07
	10^6^	3.00 ± 0.12	3.22 ± 0.12	2.97 ± 0.13	3.04 ± 0.09	2.90 ± 0.05	0.06 ± 0.37	3.14 ± 0.13
	10^7^	3.04 ± 0.15	3.08 ± 0.21	2.95 ± 0.27	2.93 ± 0.10	1.38 ± 0.10	−2.10 ± 0.29	2.99 ± 0.12
LYC1511	10^5^	2.76 ± 0.18	2.85 ± 0.12	2.88 ± 0.08	2.46 ± 0.28	3.07 ± 0.08	2.92 ± 0.18	2.93 ± 0.16
	10^6^	2.77 ± 0.08	0.78 ± 0.42	0.99 ± 0.32	−0.06 ± 0.37	2.95 ± 0.16	0.95 ± 0.34	2.87 ± 0.13
	10^7^	2.73 ± 0.23	−6.17 ± 0.00*	−6.17 ± 0.00*	−6.17 ± 0.00*	0.71 ± 0.44	−6.17 ± 0.00*	2.93 ± 0.05
**(B)**								
LYC1031	10^5^	3.19 ± 0.12	−6.17 ± 0.00*	3.06 ± 0.14	3.09 ± 0.12	−0.54 ± 0.42	−6.17 ± 0.00*	3.29 ± 0.15
	10^6^	3.11 ± 0.19	−6.17 ± 0.00*	3.06 ± 0.11	2.87 ± 0.13	−6.17 ± 0.00*	−6.17 ± 0.00*	2.87 ± 0.27
	10^7^	2.83 ± 0.20	−6.17 ± 0.00*	2.98 ± 0.27	1.94 ± 0.39	−6.17 ± 0.00*	−6.17 ± 0.00*	2.29 ± 0.21
LYC1112	10^5^	2.96 ± 0.16	−6.15 ± 0.00*	3.05 ± 0.12	2.85 ± 0.21	−0.95 ± 0.41	−6.15 ± 0.00*	2.96 ± 0.23
	10^6^	3.05 ± 0.17	−6.15 ± 0.00*	2.96 ± 0.15	2.80 ± 0.17	−1.70 ± 0.28	−6.15 ± 0.00*	2.47 ± 0.35
	10^7^	2.96 ± 0.21	−6.15 ± 0.00*	2.51 ± 0.06	1.15 ± 0.42	−6.15 ± 0.00	−6.15 ± 0.00*	2.21 ± 0.48
LYC1117	10^5^	3.03 ± 0.24	−6.15 ± 0.00*	−6.15 ± 0.00*	−6.15 ± 0.00*	0.42 ± 0.49	−6.15 ± 0.00*	3.11 ± 0.13
	10^6^	3.00 ± 0.11	−6.15 ± 0.00*	−6.15 ± 0.00*	−6.15 ± 0.00*	−6.15 ± 0.00*	−6.15 ± 0.00*	3.00 ± 0.28
	10^7^	3.08 ± 0.45	−6.15 ± 0.00*	−6.15 ± 0.00*	−6.15 ± 0.00*	−6.15 ± 0.00*	−6.15 ± 0.00*	3.25 ± 0.09
LYC1119	10^5^	3.61 ± 0.16	2.08 ± 0.22	−5.03 ± 0.00*	−5.03 ± 0.00*	3.51 ± 0.12	−5.03 ± 0.00*	4.12 ± 0.04
	10^6^	3.50 ± 0.17	−1.71 ± 0.42	−5.03 ± 0.00*	−5.03 ± 0.00*	2.37 ± 0.23	−5.03 ± 0.00*	3.56 ± 0.13
	10^7^	3.48 ± 0.23	−5.03 ± 0.00*	−5.03 ± 0.00*	−5.03 ± 0.00*	0.25 ± 0.39	−5.03 ± 0.00*	3.41 ± 0.19
LYC1146	10^5^	3.47 ± 0.13	−5.79 ± 0.00*	−5.79 ± 0.00*	−5.79 ± 0.00*	−0.38 ± 0.35	−5.79 ± 0.00*	3.11 ± 0.09
	10^6^	3.41 ± 0.22	−5.79 ± 0.00*	−5.79 ± 0.00*	−5.79 ± 0.00*	−1.61 ± 0.29	−5.79 ± 0.00*	2.59 ± 0.25
	10^7^	2.90 ± 0.19	−5.79 ± 0.00*	−5.79 ± 0.00*	−5.79 ± 0.00*	−5.79 ± 0.00*	−5.79 ± 0.00*	1.59 ± 0.44
LYC1154	10^5^	2.54 ± 0.13	2.52 ± 0.25	−6.08 ± 0.00*	−1.97 ± 0.44	2.53 ± 0.15	−6.08 ± 0.00*	3.18 ± 0.13
	10^6^	2.43 ± 0.29	−6.08 ± 0.00*	−6.08 ± 0.00*	−6.08 ± 0.00*	1.38 ± 0.24	−6.08 ± 0.00*	3.00 ± 0.12
	10^7^	2.45 ± 0.21	−6.08 ± 0.00*	−6.08 ± 0.00*	−6.08 ± 0.00*	−3.48 ± 0.19	−6.08 ± 0.00*	2.37 ± 0.37
LYC1159	10^5^	3.04 ± 0.29	0.85 ± 0.49	−5.68 ± 0.00*	−5.68 ± 0.00*	−5.68 ± 0.00*	−5.68 ± 0.00*	3.43 ± 0.10
	10^6^	3.32 ± 0.12	−5.68 ± 0.00*	−5.68 ± 0.00*	−5.68 ± 0.00*	−5.68 ± 0.00*	−5.68 ± 0.00*	3.36 ± 0.09
	10^7^	3.43 ± 0.11	−5.68 ± 0.00*	−5.68 ± 0.00*	−5.68 ± 0.00*	−5.68 ± 0.00*	−5.68 ± 0.00*	2.94 ± 0.14
LYC1229	10^5^	3.15 ± 0.07	1.33 ± 0.43	−6.05 ± 0.00*	−6.05 ± 0.00*	−6.05 ± 0.00*	−6.05 ± 0.00*	2.95 ± 0.07
	10^6^	2.66 ± 0.14	−6.05 ± 0.00*	−6.05 ± 0.00*	−6.05 ± 0.00*	−6.05 ± 0.00*	−6.05 ± 0.00*	2.52 ± 0.25
	10^7^	2.51 ± 0.30	−6.05 ± 0.00*	−6.05 ± 0.00*	−6.05 ± 0.00*	−6.05 ± 0.00*	−6.05 ± 0.00*	2.21 ± 0.28
LYC1322	10^5^	3.56 ± 0.13	−5.72 ± 0.00*	3.60 ± 0.09	3.56 ± 0.02	−0.13 ± 0.56	−5.72 ± 0.00*	3.46 ± 0.14
	10^6^	3.60 ± 0.11	−5.72 ± 0.00*	3.46 ± 0.05	2.94 ± 0.15	0.39 ± 0.72	−5.72 ± 0.00*	3.43 ± 0.15
	10^7^	3.48 ± 0.25	−5.72 ± 0.00*	3.39 ± 0.24	0.60 ± 0.44	−5.72 ± 0.00*	−5.72 ± 0.00*	2.46 ± 0.32
LYC1504	10^5^	3.08 ± 0.12	3.06 ± 0.13	3.04 ± 0.16	3.08 ± 0.12	3.02 ± 0.07	−2.58 ± 0.59	3.00 ± 0.08
	10^6^	2.92 ± 0.16	3.22 ± 0.06	3.03 ± 0.12	3.08 ± 0.06	−2.58 ± 0.36	−6.02 ± 0.00*	2.42 ± 0.21
	10^7^	2.84 ± 0.27	2.98 ± 0.15	−0.54 ± 0.45	0.46 ± 0.21	−6.02 ± 0.00*	−6.02 ± 0.00*	3.06 ± 0.14
LYC1511	10^5^	2.69 ± 0.25	3.01 ± 0.08	−6.17 ± 0.00*	−0.60 ± 0.41	2.86 ± 0.09	−6.17 ± 0.00*	2.81 ± 0.16
	10^6^	2.76 ± 0.11	−6.17 ± 0.00*	−6.17 ± 0.00*	−6.17 ± 0.00*	1.48 ± 0.21	−6.17 ± 0.00*	2.82 ± 0.20
	10^7^	2.72 ± 0.12	−6.17 ± 0.00*	−6.17 ± 0.00*	−6.17 ± 0.00*	−2.57 ± 0.42	−6.17 ± 0.00*	2.81 ± 0.19

Non, no added carbohydrate; SUC, sucrose; FOS, fructooligosaccharide; IN, inulin; IMO, isomalto-oligosaccharide; LU, lactulose; XOS, xylooligosaccharide.

* Indicates no detection of CFUs in the culture media.

#### Animal model

**Figure 3:**
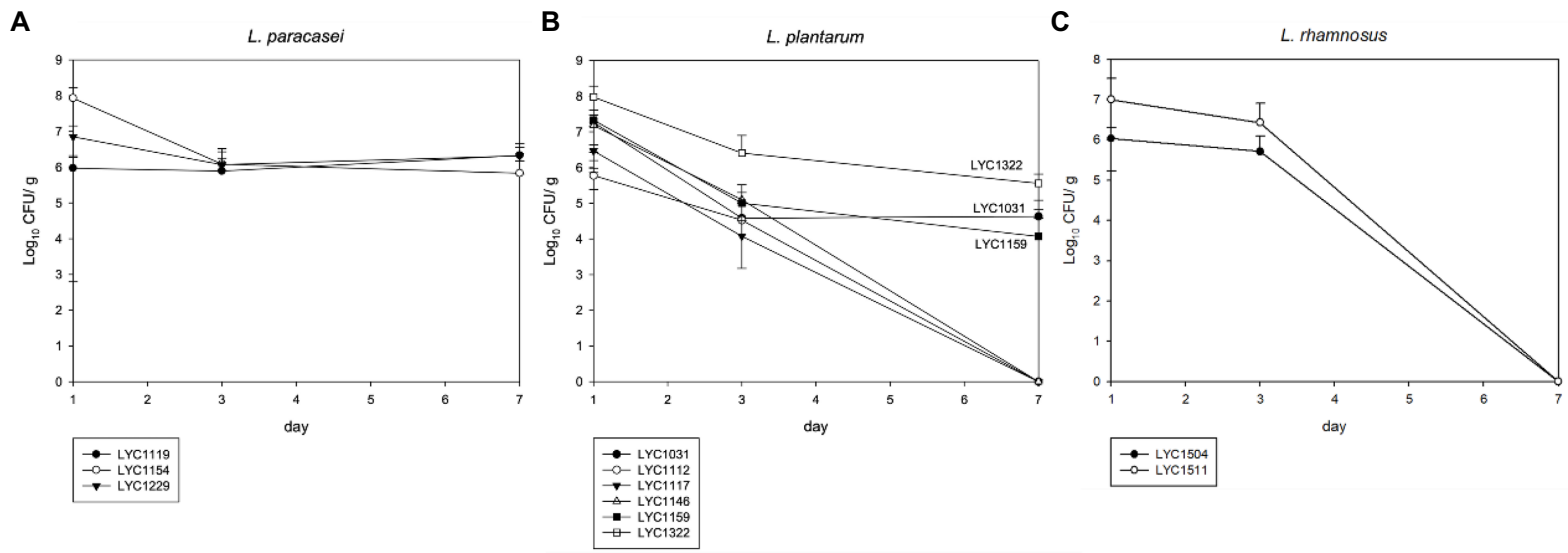
The *Lactobacillus* number in feces after each oral treatment. **(A)** *L. paracasei*, **(B)** *L. plantarum*, and **(C)** *L. rhamnosus.* (The *Lactobacillus* strains in stool are shown in CFUs/g as the mean ± SD.)

For 3 *L. paracasei* strains (LYC119, LYC1154, and LYC1229), all of them could be persistently detected, and their amount remained at ~10^6^ CFU/g in the stool on Day 7 (Figure 3A). Among 6 *L. plantarum* strains (LYC1031, LYC1112, LYC1117, LYC1146, LYC1150, and LYC1322), 4 became undetectable on Day 7, and only 3 (LYC1322, LYC1031, and LYC1159) remained detectable with amounts >10^4^ CFU/g (Figure 3B). For 2 *L. rhamnosus* strains (LYC1504 and LYC1511), both were undetectable on Day 7 (Figure 3C). Thereafter, three strains, LYC1154, 1322, and 1511, were selected for further tests against KPC-2-producing *K. pneumoniae.*

**Figure 4:**
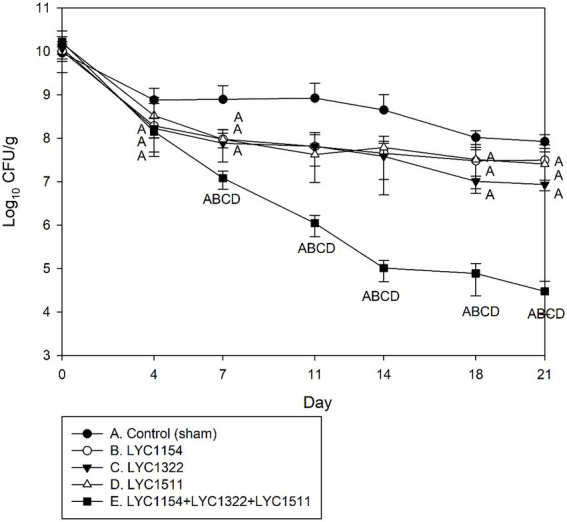
Kinetics of KPC001 from feces in treatments with one of three *Lactobacillus* strains or the three lactobacilli combined. Each symbol represents the mean ± SD of colony-forming units per gram (CFU/g) in feces from different treatment mice. (Group A: no Lactobacillus treatment, B: LYC1154, C: LYC1322, D: LYC1511, and E: LYC1154 + LYC1322 + LYC1511. The letter indicates a significant difference compared with the indicated group.)

**Figure 5:**
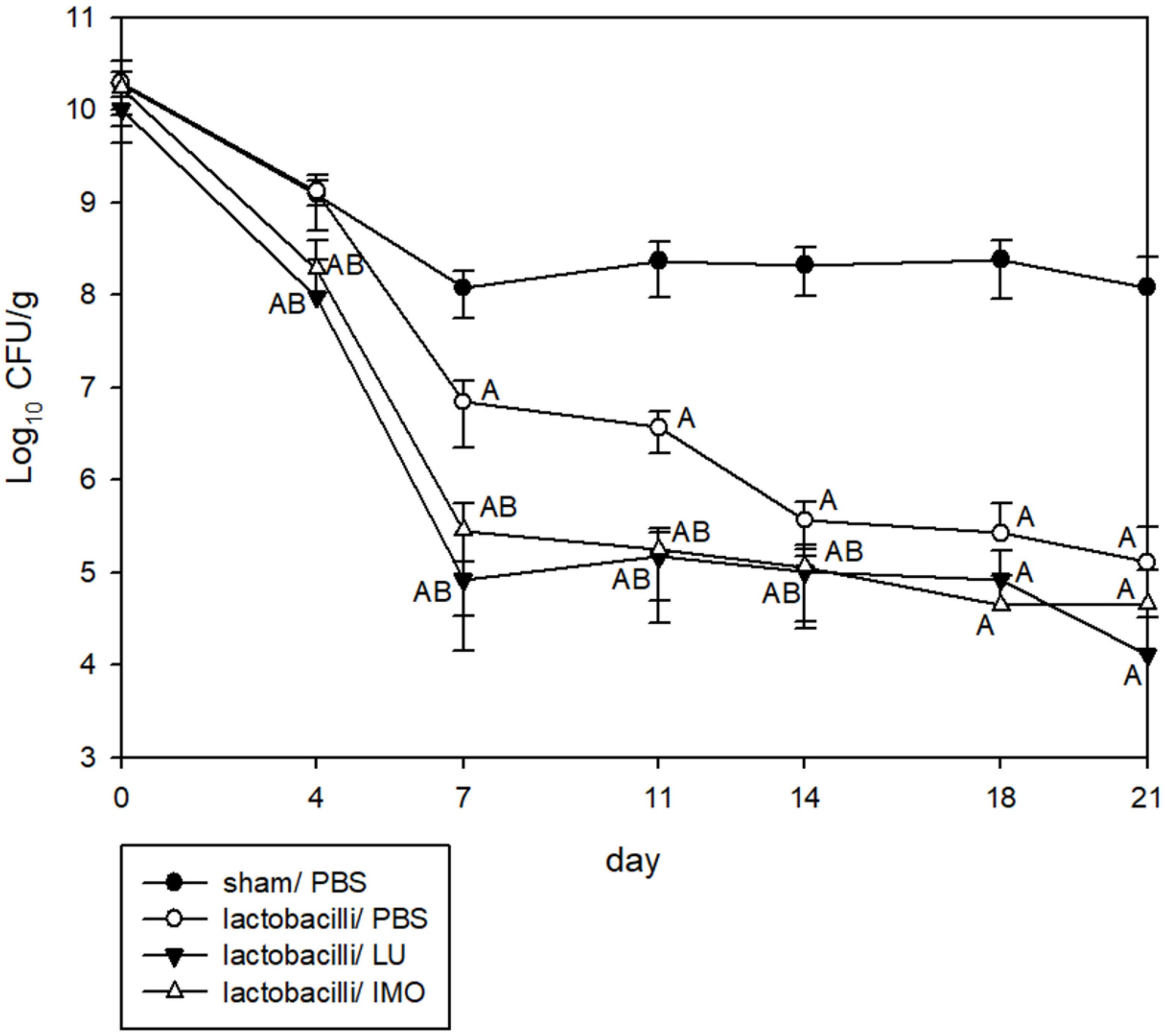
Kinetics of KPC001 from feces in three lactobacilli combined with PBS, lactulose or isomalto-oligosaccharide. Each symbol represents the mean ± SD of colony-forming units per gram (CFU/g) in feces from different treatment mice. (Group A: no lactobacilli or prebiotics, B: lactobacilli only, C: LYC1322, LYC1154, and LYC1511 with added LU, D: LYC1322, LYC1154, and LYC1511 with added IMO. LU: lactulose; IMO: isomalto-oligosaccharide. The letter indicates a significant difference compared with the indicated group.)

Three *Lactobacillus* strains (LYC1154, LYC1322, and LYC1511) were tested for their ability to reduce the amount of KPC001 in the feces individually or in combination (Figure 4). A significantly better effect in reducing the amount of KPC001 was observed for the combination of 3 different *Lactobacillus* species than for each species alone (Figure 4). Furthermore, their inhibitory effect was enhanced after adding LU or IMO (both *p* < 0.05, Figure 5).

## Discussion

This is the first study to investigate the antibacterial effect of *Lactobacillus* spp. with prebiotics against KPC-2-producing *K. pneumoniae.* From the serial tests of 33 *Lactobacillus* strains and five prebiotics, including FOS, IN, IMO, LU, and xylooligosaccharide, we identified three *Lactobacillus* strains (LYC1154, LYC1322, and LYC1511), and LU or IMO exhibited the greatest potency against KPC-2-producing *K. pneumonia.*

This finding was supported by the following evidence. First, the CSFs of the three *Lactobacillus* strains exhibited low pH values and low MIPs against the four KPC-2-producing *K. pneumoniae* strains tested (Table 1). Second, the pH values of all three strains with LU or IMO were lower than those with other prebiotics (Table 2). Third, the time-killing methods showed that both LU and IMO can help enhance the inhibitory effect of these three strains against KPC-2-producing *K. pneumoniae*—KPC001 (Table 3). Fourth, the animal model demonstrated that each of them can reduce the intestinal colonization of KPC001, and the combination of all three strains exhibited a better anti-colonization effect than each of them alone (Figure 4). Finally, after adding LU or IMO, the anti-colonization effect of the *Lactobacillus* strain against KPC001 was further enhanced in the animal experiment (Figure 5). All of these findings based on *in vitro* studies and animal models indicated the potent anti-KPC-2-producing *K. pneumoniae* of three *Lactobacillus* strains (LYC1154, LYC1322, and LYC1511) with the prebiotics LU and IMO. Moreover, Abramov et al. demonstrated that *Limosilactobacillus fermentum* strain 3872 and Actigen prebiotic (Alltech Inc., Nicholasville, KY, United States) could exhibit synergistic anti-adhesive activity against gram-negative pathogens (Abramov et al., 2022). Overall, these findings suggest the promising role of synbiotics (probiotics + prebiotics) as a new strategy for fighting MDROs.

In addition to KPC-2-producing *K. pneumoniae*, several studies (Asahara et al., 2001; Wieërs et al., 2020; Hai and Huang, 2021; Scillato et al., 2021) have reported the effect of probiotics against MDROs. Hai et al. demonstrated that the growth of MDR *Salmonella enteritidis* SE05 decreased over time by coculturing with *L. reuteri* Lb11 (isolated from the chicken intestinal tract) *in vitro*, and the pH value significantly decreased (Hai and Huang, 2021). Scillato et al. showed that the CFS of *L. gasseri* 1A-TV, *L. fermentum* 18A-TV, and *L. crispatus* 35A-TV (isolated from the vaginal microbiota of healthy premenopausal women) and their combination revealed a strong bactericidal effect on uropathogens, such as *S. agalactiae, E. coli, K. pneumoniae, S. aureus, P. aeruginosa, P. vulgaris,* and *P. mirabilis,* and MDROs, such as KPC-3-producing *K. pneumoniae* and vancomycin-resistant enterococcus (Scillato et al., 2021). Moreover, a randomized clinical trial reported that treatment with a probiotic mixture containing *Saccharomyces boulardii*, *Lactobacillus acidophilus* NCFM, *Lactobacillus paracasei* Lpc-37, *Bifidobacterium lactis* Bl-04, and *Bifidobacterium lactis* Bi-07 could significantly reduce gastrointestinal colonization with *P. aeruginosa* and AmpC-producing enterobacteria after amoxicillin-clavulanate pretreatment (*p* = 0.041). Similarly, Asaharathe et al., using an opportunistic antibiotic-induced murine infection model, showed that an increase in the concentration of organic acids and a lowered pH in the intestine due to bifidobacterial colonization were correlated with anti-infectious activity (Asahara et al., 2001). Although our findings were in line with these studies (Asahara et al., 2001; Wieërs et al., 2020; Hai and Huang, 2021; Scillato et al., 2021) and confirmed the inhibitory effect of probiotics against MDROs, our finding further indicated that multiple probiotics in combination with prebiotics would have greater activity.

However, one RCT found that the administration of a synbiotic product twice a day for 7 days *via* the oral/enteral route was not effective for decolonizing hospitalized patients harboring MDR gram-negative bacilli (Salomão et al., 2016). In this single-center study of 116 patients, no significant difference was observed in the negative recovery rate of rectal swabs for MDR gram-negative bacilli after treatment between the synbiotic group and the placebo group (16.7% [8/48] vs. 20.7% [11/53], *p* = 0.60) (Salomão et al., 2016). The possible explanation for these conflicting findings between the RCT and the present study could be the different study designs—*in vitro* or *in vivo* vs. human study. In addition, we identified three *Lactobacillus* strains (LYC1154, LYC1322, and LYC1511) and two prebiotics, LU and IMO, as exhibiting the greatest activity, but the RCT used the synbiotics *Lactobacillus bulgaricus, Lactobacillus rhamnosus*, and FOSs. Therefore, further study is needed to clarify our findings.

This study has two limitations. First, as we only focused on KPC-2-producing *K. pneumoniae*, our findings cannot be generalized to other MDROs. Second, the composition and percentage of the three lactobacilli and prebiotics were not clearly defined in the study. Further *in vivo* studies are warranted.

In conclusion, our study strengthens the concept of using probiotic *Lactobacillus* with prebiotics to protect the host against the MDR pathogen KPC-2-producing *K. pneumoniae*, and these results support novel therapeutic strategies as new synbiotics for the prevention and treatment of MDRO colonization or infections.

## Data availability statement

The raw data supporting the conclusions of this article will be made available by the authors, without undue reservation.

## Author contributions

H-JT, C-CC, C-CL, and C-MC contributed to conception and design of the study. H-JT, C-CC, Y-CL, H-LH, H-JC, Y-CC, C-CL investigation, analysis, or interpretation of data for the work. C-CC, Y-CL, H-LH, H-JC, performed the statistical analysis. H-JT, C-CL, Y-CC, C-MC wrote the first draft of the manuscript. H-JT and C-MC critical review. All authors contributed to manuscript revision, read, and approved the submitted version.

## Funding

This study was supported by a research grant (MOST 111-2314-B-384-008) from the Ministry of Science and Technology and the Chi-Mei Medical Center Research Foundation (CMFHR11067 and CMFHT11001).

## Conflict of interest

The authors declare that the research was conducted in the absence of any commercial or financial relationships that could be construed as a potential conflict of interest.

## Publisher’s note

All claims expressed in this article are solely those of the authors and do not necessarily represent those of their affiliated organizations, or those of the publisher, the editors and the reviewers. Any product that may be evaluated in this article, or claim that may be made by its manufacturer, is not guaranteed or endorsed by the publisher.
